# High-Frequency Plasma Electrolytic Oxidation of an Al–Si Alloy: Influence of Al_2_O_3_ and SiO_2_ Additives on Coating Microstructure and Tribological Performance

**DOI:** 10.3390/ma18235334

**Published:** 2025-11-26

**Authors:** Gulzhaz Uazyrkhanova, Amangeldi Sagidugumar, Yernat Kozhakhmetov, Gulzhaz Moldabayeva, Daniyar Kaliyev, Sergey Rudenko, Nurgamit Kantay

**Affiliations:** 1 The Center of Excellence “VERITAS”, D. Serikbayev East Kazakhstan Technical University, Ust-Kamenogorsk 070004, Kazakhstan; 2 The Competence Center «Smart Engineering», D. Serikbayev East Kazakhstan Technical University, Ust-Kamenogorsk 070004, Kazakhstan; 3 The National Scientific Laboratory of Collective Use, Sarsen Amanzholov East Kazakhstan University, Ust-Kamenogorsk 070020, Kazakhstan

**Keywords:** Al–Si alloys, plasma electrolytic oxidation, tribology, wear, Al_2_O_3_, SiO_2_

## Abstract

This study focuses on surface modification of aluminum alloys (Al–Si) with high silicon content using plasma electrolytic oxidation (PEO). The influence of Al_2_O_3_ and SiO_2_ particles, introduced both separately and in combination, into a sodium aluminate-based electrolyte during high-frequency treatment (2000 Hz). Examination of surface and cross-sections using a scanning electron microscope SEM showed an increase in the compactness of the coating when Al_2_O_3_ particles were introduced. The addition of SiO_2_ particles tended to promote a smoother surface and a slight reduction in the porosity and defect density. However, when these particles are added together, especially at high concentrations, an increase in structural defects and crack formation is observed. X-ray diffraction analysis revealed that the γ-Al_2_O_3_ phase was present in all coatings. In the samples with Al_2_O_3_ addition, the α-Al_2_O_3_ diffraction signal became stronger compared with the other coatings. Tribological tests revealed that the addition of Al_2_O_3_ particles significantly improved wear resistance, while the introduction of SiO_2_ particles contributed to the stabilization of the friction coefficient. Thus, Al_2_O_3_ particles were the most effective in enhancing the mechanical properties of the coating.

## 1. Introduction

Foundry aluminum alloys with high silicon content (Al–Si) are widely used in various industries, including aviation, shipbuilding, and automotive. In the automotive industry, there is a need to use new materials, especially in engine parts, to reduce vehicle weight and consequently reduce fuel consumption, which contributes to lower greenhouse gas emissions [1,2,3]. The use of aluminum alloys is often limited by their relatively low tribological and mechanical properties. Currently, there is a great deal of research aimed at modifying foundry aluminum alloys in order to improve their performance characteristics [4]. One of the promising modification methods is plasma electrolytic oxidation (PEO), which allows the formation of hard and wear-resistant ceramic coatings on the alloy surface [5,6,7].

However, PEO technology has certain disadvantages associated with the formation of defects in the coating structure. PEO coatings are characterized by high porosity, voids, microcracks, and non-uniform thickness. Such defects can reduce the mechanical properties of the formed layers. Pores and cracks often act as stress concentrators and reduce the adhesion strength of the coating, which can ultimately lead to premature failure. Defect formation in PEO coatings is primarily related to the high energy density released in microdischarges and their instability [8,9]. Optimization of the electrical mode makes it possible to reduce the number of such defects. A number of studies [10,11,12] suggest the use of the high-frequency mode of PEO (>1 kHz), which provides softer and more stable microdischarges, which promotes the formation of smoother coatings with fewer defects.

Moreover, composite coatings are widely used by introducing functional nano- and micro-particles into the electrolyte to compensate for defects in PEO layers and improve tribological properties. For example, solid ceramic particles such as Al_2_O_3_, SiO_2_, ZrO_2_, and TiO_2_ are introduced to increase the coating’s hardness and wear resistance [13]. The graphite or h-BN (hexagonal boron nitride), such as solid lubricant particles, provides self-lubricating properties and significantly reduces the coefficient of friction [14]. The most effectiveness of PEO coatings is achieved through hybrid or duplex methods. Such methods combine the PEO process with additional modifications, allowing for the reduction in defects in the coating and the optimization of its mechanical properties. One such treatment is pre-mechanical hardening, for example, SMAT (Surface Mechanical Attrition Treatment) [4]. This method induces compressive residual stresses on the metal surface, reducing the negative impact of the PEO layer’s porosity and microcracks on the alloy’s fatigue strength. Also, subsequent treatment methods—sealing—are widely used. In this method, the pores of the PEO layer are filled with special polymers. One of the most commonly used materials is PTFE (Polytetrafluoroethylene), as well as epoxy resins [14,15]. Filling the pores with such polymers significantly increases the coating’s resistance to corrosion and wear, densifies its structure, and enhances its suitability for aggressive operating conditions. Current literature shows that PEO coatings provide good hardness and wear resistance, but their porous structure and microcracks limit full tribological protection. Therefore, complementary methods such as composite modification, pre-hardening, or sealing are often required to further enhance the performance of PEO layers.

In addition, the composition of the electrolyte and the introduction of ceramic particles have a significant influence on the quality of the coating in the PEO process. In several studies [1,16], it is noted that electrolytes based on sodium aluminate (NaAlO_2_) are the most effective for aluminum alloys with high silicon content. The addition of Al_2_O_3_ particles in such electrolytes contributes to the increase in coating thickness and the formation of the α-Al_2_O_3_ phase, which leads to an increase in its mechanical properties [13,17]. At the same time, the introduction of SiO_2_ particles contributes to the reduction in pore sizes and the number of defects, as well as the formation of a smoother surface, which improves the tribological characteristics of the coating and increases its wear resistance [18].

The main objective of this work lies in the systematic comparison of Al_2_O_3_ and SiO_2_ particles—used individually and in combination—in a NaAlO_2_-based electrolyte under a high-frequency PEO regime (2000 Hz), allowing for the first time to distinguish their separate and synergistic effects on morphology, phase evolution, and tribological performance of a high-Si aluminum alloy.

## 2. Materials and Methods

### 2.1. Substrate Preparation

Disks with a 22 mm diameter were obtained from an A333 Al–Si alloy to serve as the substrate. The alloy’s composition was 84.55% Al, 10.72% Si, 2.22% Cu, 0.43% Mg, 0.18% Mn, 0.91% Fe, and 0.74% Zn. The substrate surfaces were first sequentially ground with silicon carbide (SiC) sandpaper of 250, 500, 1000, and 2000 grit. Subsequently, they were polished with a 3-µm diamond suspension to achieve a mirror finish. Although this treatment is not mandatory for the PEO process, it was applied to standardize the initial surface roughness of all samples to the same level in order to eliminate the influence of the initial surface condition in tribological tests. Standardizing the initial surface allowed the differences in coating properties to be attributed solely to the effect of the PEO process and electrolyte composition. Finally, the substrates were cleaned in distilled water using an ultrasonic bath for 10 min and then dried at room temperature.

### 2.2. Formation of PEO Coatings

The prepared samples were treated by PEO in an aluminate electrolyte (24 g/L NaAlO_2_ + 1 g/L KOH) containing various concentrations of Al_2_O_3_ and SiO_2_ powders, which were added either separately or in combination. The powders used in this study were first characterized using a laser particle-size analyzer (Malvern Mastersizer (Malvern Panalytical, Malvern, UK)). The Al_2_O_3_ powder (Bengbu Zhongheng New Materials Co., Ltd., Bengbu, China) exhibited a micro-sized distribution with an average particle size of 1.768 µm (D [4,3] = 1.66–1.78 µm) and a modal value of 1.7–1.8 µm, corresponding to a polyhedral, microcrystalline morphology typical for fine α-Al_2_O_3_. The SiO_2_ powder (Shijiazhuang Haocheng New Material Technology Co., Ltd., Shijiazhuang, China) showed a coarse micro-sized distribution with an average particle size of 14.046 µm (D [4,3] = 10.24–10.67 µm) and a modal value of 13–15 µm, consistent with spherical to sub-spherical amorphous silica. The electrolyte compositions are summarized in Table 1. The aluminate electrolyte composition was selected based on the works presented in [16,19], where the formation of single-layer PEO coatings with increased tribological properties was reported. In addition, the concentrations of the Al_2_O_3_ and SiO_2_ powders were selected based on the results reported by Huang et al. [20] and Ghafaripoor et al. [17].

**Table 1:** The electrolyte compositions.

	Electrolyte Composition	NaAlO_2_ (g/L)	KOH (g/L)	Al_2_O_3_ (g/L)	SiO_2_ (g/L)
E1	Base electrolyte	24	1	–	–
E2	Base + SiO_2_	24	1	–	3.0
E3	Base + Al_2_O_3_	24	1	3.0	–
E4	Base + Al_2_O_3_ + SiO_2_ (1 g/L each)	24	1	1.0	1.0
E5	Base + Al_2_O_3_ + SiO_2_ (2 g/L each)	24	1	2.0	2.0
E6	Base + Al_2_O_3_ + SiO_2_ (3 g/L each)	24	1	3.0	3.0

**Figure 1:**
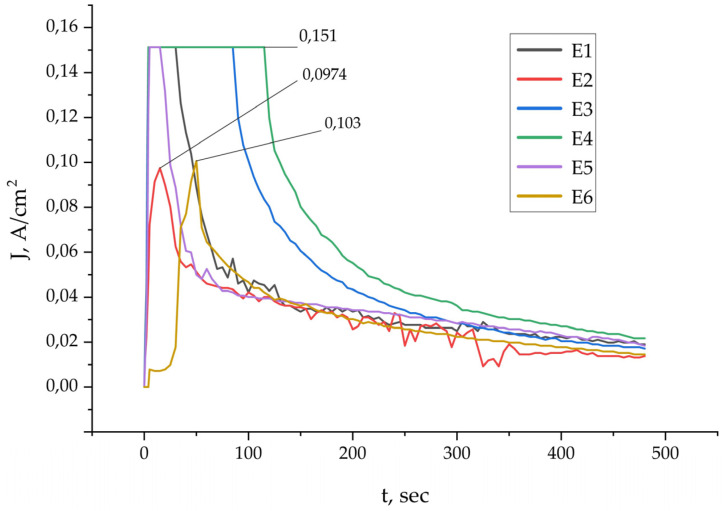
Current density versus time for electrolytes E1–E6 during PEO processing. Differences between the curves reflect the effect of Al_2_O_3_ and SiO_2_ particle additions in each electrolyte.

The obtained samples were treated by plasma electrolytic oxidation (PEO) using a DC pulse power supply (Model NHTW-TG-50-40, Jinan Nenghua Mechanical and Electrical Equipment Co., Ltd., Jinan, China; website: www.nenghua.com.cn). The device provides an adjustable output voltage range of 0–750 V, a maximum output current of 40 A, and an operating frequency range of 50–3000 Hz.

The PEO processing parameters were selected based on data reported in previous studies and established physical principles of the technique. The electrical regime—350 V, 2000 Hz, a 20% duty cycle, and a maximum current of 2.3 A—ensured the formation of stable, low-energy discharges in a unipolar pulsed mode, thereby reducing the number of coating defects [10,12]. The treatment time was set to 8 min to ensure uniform layer growth while preventing overheating and cracking. The electrolyte temperature during the PEO process was maintained at approximately 25–30 °C. Additionally, a preliminary 1 min treatment in a 2 g/L NaAlO_2_ solution was performed to prevent field-assisted dissolution in Al–Si alloys [16,21]. Thus, all process parameters were determined based on literature data, the physics of the PEO process, and the characteristics of the alloy.

During PEO, the current density varied dynamically depending on time and electrolyte composition. In the initial stage, a rapid increase in current was observed, corresponding to the breakdown of the native oxide film and the onset of plasma discharges [8,22]. Subsequently, the current gradually decreased and stabilized as the oxide layer formed steadily. The particles present in the electrolyte had a pronounced effect on the current behavior: in electrolytes containing SiO_2_, the current density was lower due to increased electrical resistance, whereas in systems with Al_2_O_3_, the discharge intensity and current values slightly increased [17,20]. This dependence is illustrated in Figure 1.

The experimental setup consisted of a stainless-steel container with a cooling system, which served as the cathode. The Al–Si sample, immersed in the electrolyte, served as the anode.

### 2.3. Coating Analysis

The surface morphologies and cross-sections of the PEO coatings were analyzed using a Tescan vega 4 Scanning Electron Microscope (SEM) (TESCAN Orsay Holding, Brno, Czech Republic). The SEM was also equipped with an Oxford Xplore 30 Energy Dispersive Spectrometer (EDS) (Oxford Instruments, High Wycombe, UK), which was used to determine the elemental composition of the coatings. The phase composition was characterized by X-ray diffraction (XRD) using a Rigaku SmartLab diffractometer (Rigaku Corporation, Tokyo, Japan). The measurement parameters were as follows: Cu-Kα radiation (λ = 1.5406 Å); a voltage of 40 kV and a current of 50 mA; a 2θ scan range of 20° to 70°; a step size of 0.04°; and a time per step of 1 s.

Tribological tests were performed on a TRB^3^ tribometer (Anton Paar Srl, Buchs, Switzerland) using a ball-disk setup under a 5 N normal load and at a sliding speed of 5 cm/s for a total distance of 100 m. A 100Cr6 steel ball (Ø 6 mm, hardness ~60 HRC) was used as the counterbody. This material was selected because 100Cr6 steel is a standard counterpart widely used in tribological studies and mechanical engineering application [23]. The mean roughness of the polished surface of the ball was approximately Ra ≈ 0.02 µm, which ensured stable and repeatable contact conditions during sliding. After testing, the wear rates of the coatings were calculated from wear track profiles measured by a Surtronic S128 profilometer (Taylor Hobson Ltd., Leicester, UK). The initial surface roughness of the PEO coatings was also measured using the same profilometer.

Hardness measurements were conducted with the Vickers method (HV) on an automated microhardness tester, the DuraScan-20, produced by (EMCO-TEST Prüfmaschinen GmbH, Kuchl, Austria), employing a pyramidal diamond indenter. The test load was 500 gf, which corresponds to the designation HV0.5.

## 3. Results and Discussion

### 3.1. Analysis of PEO Coatings

**Figure 2:**
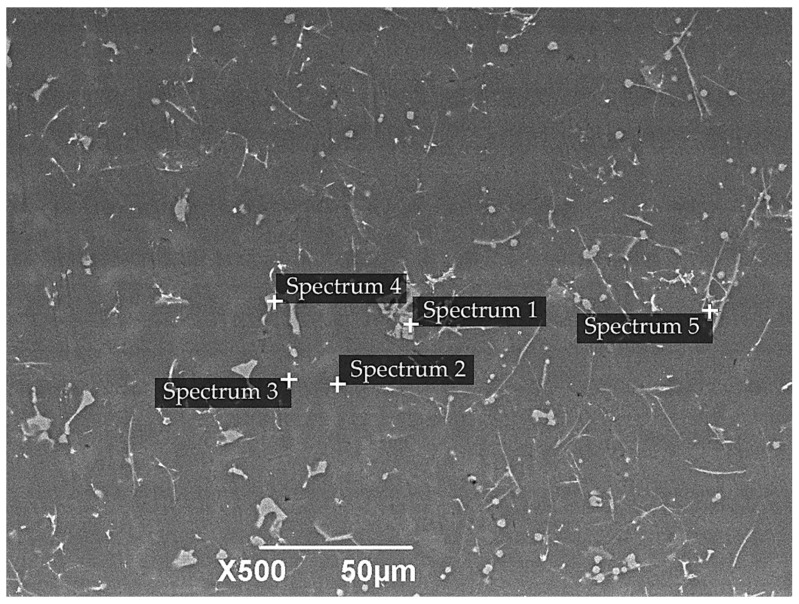
SEM image of the Al–Si substrate; the inset shows the EDS analysis (spectrum) obtained from the marked point on the microstructure.

Figure 2 illustrates the surface morphology of the substrate, which is composed of an aluminum-based solid solution (α-Al) that appears as a dark gray matrix. The eutectic silicon (Si) appears as elongated light gray inclusions within this matrix. The intermetallic compounds appear as bright white, compact particles of round or polyhedral shape [19,24]. This heterogeneous structure is characteristic of hypereutectic Al–Si alloys. The results of EDS analysis of the substrate (Table 2) show that the dominant element is aluminum, the content of which varies from 58.8 to 93.9 wt.%. In the Al–Si alloy, the distribution of silicon is uneven, so in EDS analysis the Si content can vary from approximately 2 to 28 wt.% depending on the analyzed region. The high value in Spectrum 3 is explained by measurements taken over Si-rich eutectic regions or over original silicon particles. The presence of intermetallic phases Al-Fe-Si and Al-Cu-Mn is confirmed by the presence of Fe (up to 19.6 wt.%), Cu (up to 2.1 wt.%), and Mn (up to 6.2 wt.%). A small amount of oxygen (~2–3 wt.%) indicates the formation of a thin natural oxide film on the alloy surface.

This heterogeneous microstructure of the substrate plays an important role in the subsequent PEO process. Silicon-rich phases, due to their low electrical conductivity, hinder the formation of stable microdischarges and locally reduce the coating growth rate by concentrating breakdown at phase boundaries. Meanwhile, alloying elements such as Cu and Mn can participate in electrochemical reactions during PEO and may be incorporated into the coating in the form of mixed oxides or inclusions. Thus, the microstructural features revealed by EDS analysis directly influence discharge behavior and the final morphology of the PEO coating.

**Figure 3:**
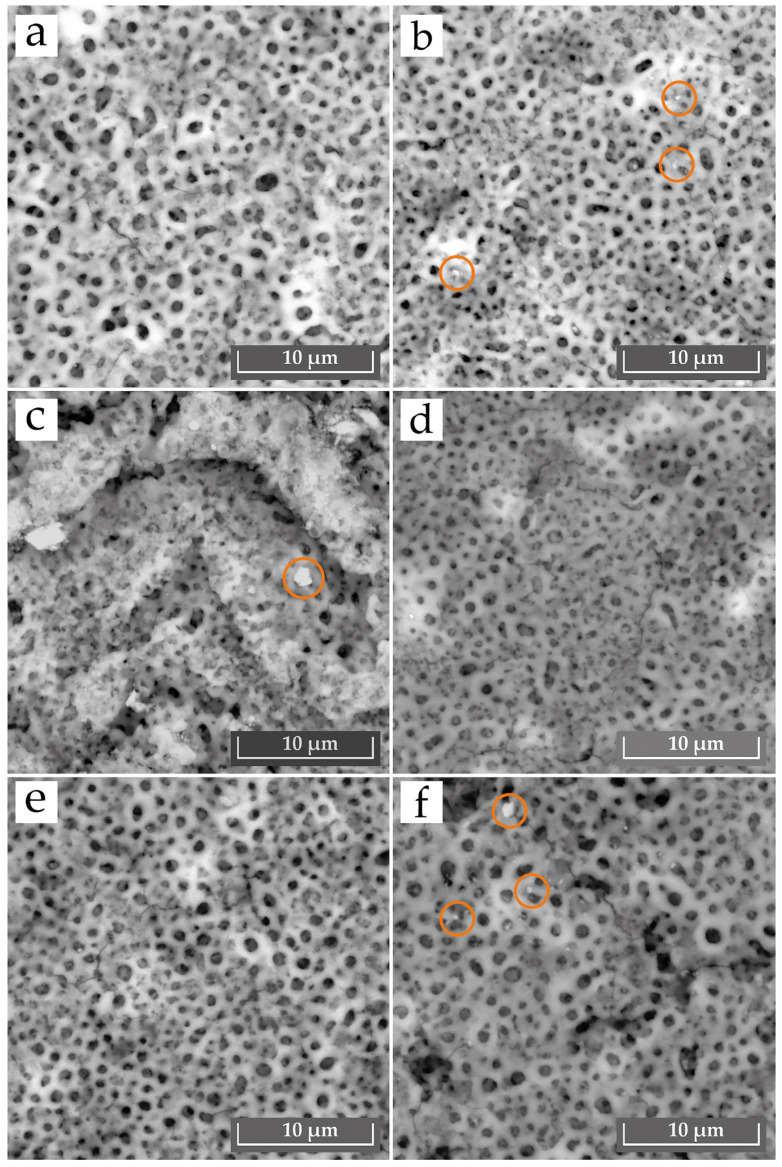
SEM images showing the surface morphology of PEO coatings: (**a**) E1, (**b**) E2, (**c**) E3, (**d**) E4, (**e**) E5, and (**f**) E6. The marked regions indicate areas where SiO_2_ or Al_2_O_3_ particles were incorporated into the coating structure.

Figure 3 illustrates the surface morphology of the PEO coatings obtained on the substrates. The coatings exhibit a porous structure, a typical feature that results from the microdischarges occurring during the PEO treatment. The coating produced in the base electrolyte (E1) exhibits relatively large, rounded pores distributed throughout its structure (Figure 3a). The structure resembles a solidified melt containing noticeable pores formed by gas emission. The PEO coating obtained in the SiO_2_-containing electrolyte (E2) has a morphology generally similar to the base coating (Figure 3b). However, compared to sample E1, two noticeable tendencies can be observed: the pores appear slightly smaller in diameter, and the surface becomes somewhat smoother. These changes may be associated with more stable plasma discharges during the PEO process, as the addition of SiO_2_ tends to lower the current density (as shown previously in Figure 1). The introduction of SiO_2_ powder increases the electrical resistance of the electrolyte-coating system. This leads to a higher breakdown voltage and promotes a greater number of smaller, more stable discharges [20]. The surface shows the presence of incorporated SiO_2_ and Al_2_O_3_ particles, as indicated by the marked areas in Figure 3. The SiO_2_ additions appear as relatively rounded inclusions (Figure 3b), whereas the Al_2_O_3_ particles exhibit a more angular shape consistent with their measured morphology (Figure 3c).

The coating from the Al_2_O_3_-containing electrolyte (E3) exhibits a morphology that is significantly different from the other samples (Figure 3c). Clumped agglomerates dominate the surface morphology, partially obscuring the underlying porous structure. This suggests that while Al_2_O_3_ particles from the electrolyte were actively incorporated into the PEO coating, some of them also formed a heterogeneous, agglomerated outer layer [17]. The coatings obtained in electrolytes E4 showed a relatively smooth and visually more homogeneous surface, with small and more uniformly distributed pores (Figure 3d). However, despite this smooth appearance, small cracks were also observed. The coating from electrolyte E6 (Figure 3f) was less homogeneous and exhibited large cracks. This can be attributed to particle agglomeration, which likely caused discharge instability and localized overheating. These conditions would exacerbate the thermal stresses from rapid solidification, leading to the observed cracking [25].

**Figure 4:**
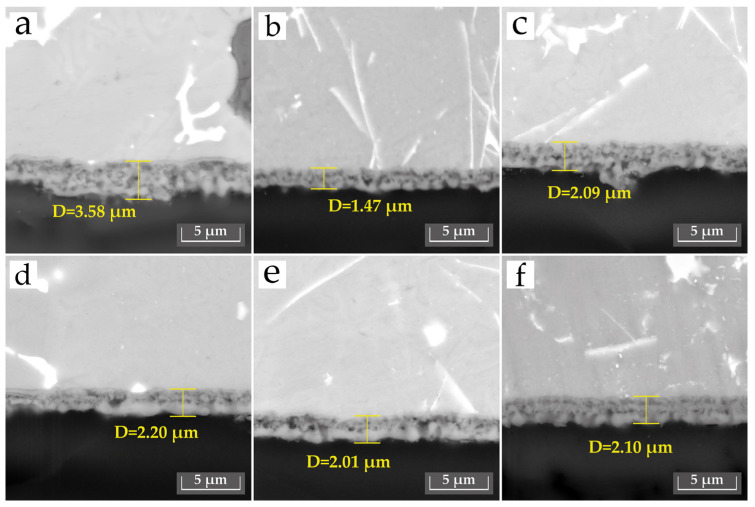
SEM images showing the cross-section of PEO coatings formed in different electrolytes: (**a**) E1, (**b**) E2, (**c**) E3, (**d**) E4, (**e**) E5, and (**f**) E6.

Figure 4 shows the cross-sectional SEM images of the PEO coatings. The coating produced in the base electrolyte (E1) is characterized by a duplex structure with a dense inner layer and a less compact outer layer, and has an overall thickness of approximately 3.5 µm. For comparison, the coating formed in the electrolyte containing SiO_2_ (E2) exhibits a more homogeneous morphology but has a lower overall thickness of approximately 1.5 µm, though some microdefects can still be observed. This reduction in coating thickness is likely associated with the decrease in current density during the PEO process.

The coating obtained with the addition of Al_2_O_3_ (E3) is shown in Figure 4c; it exhibits a more uniform morphology with reduced porosity and improved layer homogeneity, although some defects are still visible. The introduction of Al_2_O_3_ led to a slight increase in coating thickness compared with the SiO_2_-containing sample (E2), with values reaching approximately 2.0–2.2 μm. However, the coatings obtained in this study generally have a lower thickness and roughness compared to those reported in other works [3,19]. These relatively low thickness and roughness values may be attributed to the high-frequency PEO process used (2000 Hz), which promotes smaller, more stable microdischarges and leads to slower coating growth compared to conventional low-frequency regimes [22,26,27].

For the coatings obtained in the composite electrolytes (E4, E5, and E6), the number of voids and defects increased—particularly in the E5 coating (Figure 4e)—and the overall structure appears less compact. As mentioned above, this behavior can be explained by several factors. The addition of SiO_2_ and Al_2_O_3_ particles, which are electrical insulators, modifies the conductivity of the electrolyte. Furthermore, an excessive number of particles or their agglomeration may block discharge channels, resulting in a voltage rise and the occurrence of unstable, high-energy discharge [18,28]. Finally, the presence of SiO_2_ particles can inhibit the formation of the hard α-Al_2_O_3_ phase [18,20], which may also contribute to the lower compactness of the coatings. It should also be noted that the coatings obtained in E4–E6 exhibit nearly the same thickness (≈2.0–2.2 µm), so the differences observed between these samples are mainly related to structural compactness rather than layer thickness. Moreover, the cross-section indicates a variety of Al_2_O_3_ and SiO_2_ particles and also structures outgrowths, particularly in Figure 4a,c. The appearance of these outgrowths is likely due to increased currents, which locally increased discharge density, resulting in the partial manifestation of such formations.

**Figure 5:**
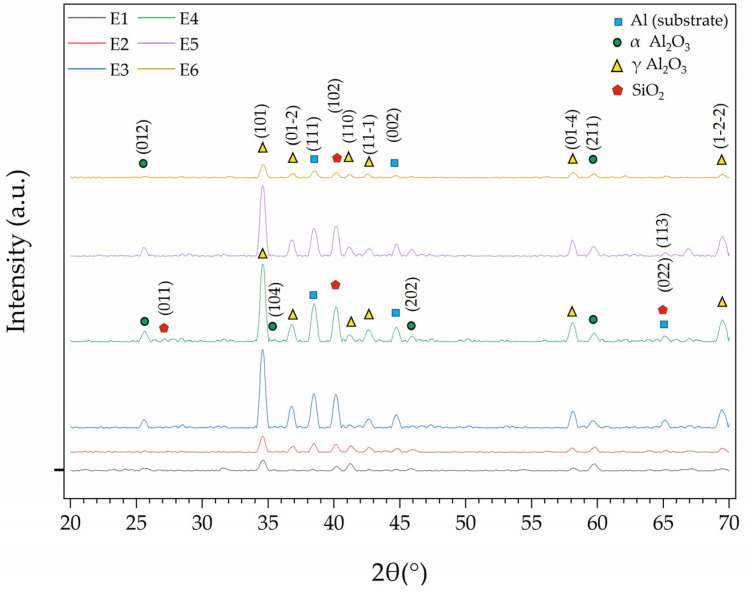
XRD patterns of the PEO coatings.

X-ray diffraction (XRD) measurements were carried out to identify the phases present in each PEO coating, and the resulting patterns are illustrated in Figure 5. In all samples, diffraction peaks corresponding to the Al substrate (ICSD 98-004-3423) were identified at the (111), (002), and (022) planes, due to the penetration of X-rays through the porous coatings.

The primary phase formed in the coatings was γ-Al_2_O_3_ (ICSD 98-017-3014), which is typical for PEO on aluminum [29,30]. Its presence was confirmed by characteristic diffraction peaks for the (101), (01-2), (110), (11-1), (01-4) and (1-2-2) planes. The more stable α-Al_2_O_3_ phase (ICSD 98-004-3732) was also detected, primarily in samples containing Al_2_O_3_ powder. For instance, peaks corresponding to α-Al_2_O_3_ at the (012), (104), (202), and (211) planes appeared in samples E3, E4, and E5.

The addition of SiO_2_ powder also had a clear effect. Crystalline SiO_2_ peaks (ICSD 98-020-0727) at the (011), (102), and (113) planes were present in all samples, but their intensity increased noticeably for the E4 and E5 coatings.

### 3.2. Tribological Tests

**Figure 6:**
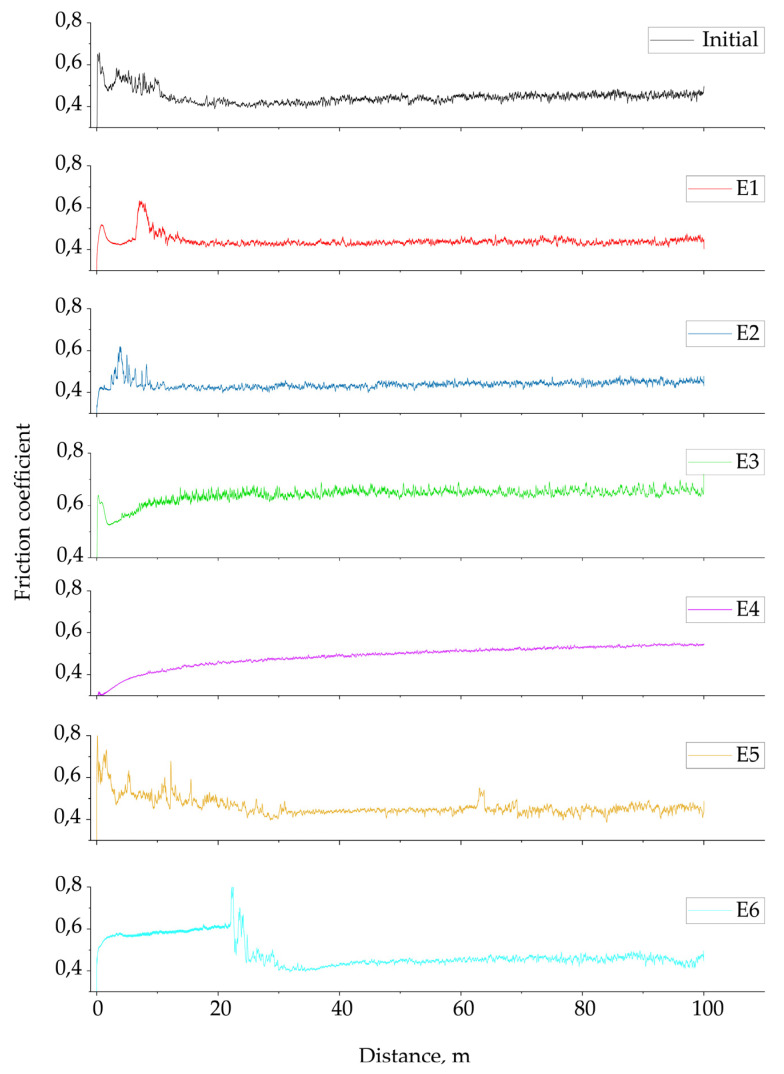
Coefficient of friction of the PEO coatings measured during the ball-on-disk tribological test.

The tribological characteristics of the PEO coatings and the initial substrate were investigated using a ball-disk tribometer. The specific test parameters are detailed in Section 2.3. The behavior of the coefficient of friction (COF) for the PEO coatings and the initial substrate under the wear test conditions is shown in Figure 6. The COF curves showed significant differences in the tribological behavior of the samples. The coating from electrolyte E3, in particular, stands out, as it demonstrated a significantly higher coefficient of friction that stabilized at an average value of 0.640 after the first ~10 m of sliding. This behavior correlates with its unique morphology, which includes its higher surface roughness, greater thickness, and the presence of crystalline Al_2_O_3_ phases [25,31].

On the other hand, the remaining coatings and the uncoated substrate all exhibited similarly low coefficients of friction. Furthermore, all friction curves exhibit a distinct initial peak, whose magnitude and position vary depending on the electrolyte composition. The initial substrate shows a peak of ~0.65 at a sliding distance of ~0.5 m. For the coated samples, the peak values occurred at different distances: E1 demonstrated a peak of ~0.62 at ~7 m, E2 a peak of ~0.61 at ~3.8 m, and E3 a peak of ~0.61 at ~3.9 m. The E4 coating exhibited a lower peak of ~0.30 at ~0.5 m. The highest and earliest peak was observed in E5, which reached ~0.80 at ~0.1 m. Finally, sample E6 showed a distinct peak of ~0.80 at a sliding distance of ~22 m. These differences in peak position and height reflect the initial instability of the tribological contact before the COF curves stabilize in the steady-state regime. After this short running-in period, the COF values of the remaining samples stabilized and remained nearly constant within the 0.44–0.49 range. Within this group, the coatings obtained from electrolytes E5 and E6 showed slightly higher and less stable coefficient of friction (COF) values compared to those from E1 and E2, which may be due to increased defect density, porosity of the oxide layer, or the presence of microcracks. Sample E4 showed an intermediate behavior; its COF steadily increased from 0.35 to 0.52 over the entire sliding distance. The stability of the COF curve suggests the formation of a homogeneous structure; however, the coating did not show improved wear resistance. This may be attributed to the combination of its limited thickness and the lower compactness of the layer.

**Figure 7:**
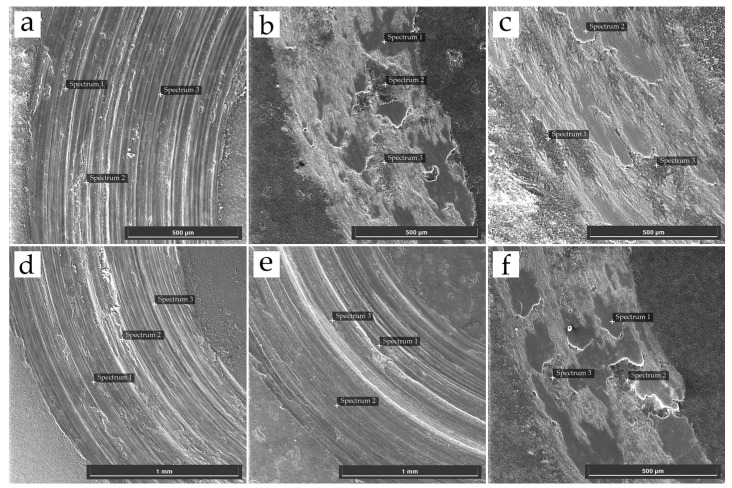
SEM images of the wear tracks after the tribological test for (**a**) E1, (**b**) E2, (**c**) E3, (**d**) E4, (**e**) E5, and (**f**) E6; the inset in each image shows the corresponding EDS spectrum obtained from the marked analysis area.

Figure 7 shows the SEM images of the wear tracks on the PEO coatings after the tribological tests. Table 3 summarizes the results of the local EDS analysis for the points indicated in these images. Samples obtained in the base electrolyte (E1) and in the composite electrolytes with low to medium powder concentrations of Al_2_O_3_ and SiO_2_ (E4, E5) showed signs of predominantly abrasive wear and coating failure. The EDS analysis of the wear track on sample E1 revealed only elements from the coating and substrate (Al, O, Si), with no iron (Fe) detected from the counterbody. Complete abrasion of the PEO coating was not observed, since a significant amount of oxygen from the oxide layer was still detected in the wear track. Samples E4 and E5 exhibited more intense degradation, as EDS analysis of their wear tracks revealed a high aluminum content and a low oxygen content Table 3. This clearly indicates the complete abrasion of the PEO layer in these areas and the exposure of the aluminum substrate.

The wear mechanism of the coating formed in the E3 electrolyte (with only Al_2_O_3_ added) is significantly different. The wear track in Figure 7c does not have distinct grooves but is instead covered by a dense, dark tribolayer. EDS analysis of this layer reveals the nature of the process: its composition is dominated by iron, whose concentration reaches 59.18 wt.%, while the aluminum content drops to 2.87 wt.% (Table 3). This indicates that intensive adhesive wear took place, involving mass transfer from the 100Cr6 steel counterbody to the PEO coating. This behavior can be attributed primarily to the presence of the hard α-Al_2_O_3_ phase and the significantly increased surface roughness of the coating. These factors enhanced the real contact area and local contact pressures, which likely promoted partial disruption of the oxide surface and micro-welding with the counterbody, leading to adhesive wear [32,33,34].

The coatings obtained in electrolyte E2 (with SiO_2_ addition) and E6 (with the maximum powder concentration) exhibit a mixed wear mechanism. Their wear tracks are visually similar to those on sample E1 and are characterized by abrasive grooves. However, unlike E6 and E2, EDS analysis detected a small but stable presence of iron (up to 0.89 wt.%) transferred from the counterbody. This suggests that the dominant abrasive wear in these coatings is accompanied by a minor adhesive wear component.

**Figure 8:**
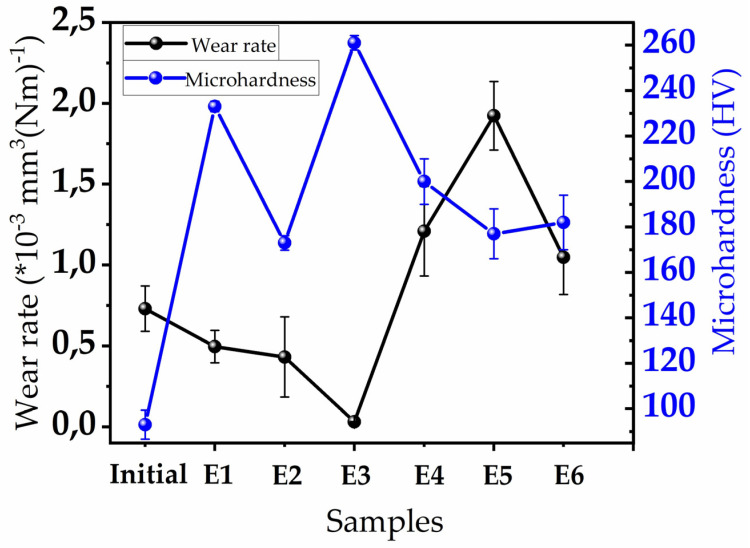
Variation in wear rate and microhardness of the PEO coatings compared to the initial substrate.

The wear rate–hardness (Figure 8) results showed that the wear resistance of PEO coatings is closely related to their phase composition. The softness of the aluminum matrix in the initial sample (93 HV) explains its high wear rate. In samples E1 and E2, the PEO process formed a dense oxide layer, significantly increasing the microhardness compared to the initial sample (to ≈230 HV and 173 HV), which contributed to improved wear resistance. 

The most effective result was observed in sample E3: the wear rate decreased to 0.030 × 10^−3^ mm^3^/N·m, and the dense structure of the coating, together with its increased microhardness (261 HV), significantly improved its overall tribological performance. In this sample, the signs of α-Al_2_O_3_ phase formation were more pronounced according to the XRD results, and its high hardness contributed to an increase in the coating’s strength and wear resistance.

In the E4–E6 samples, the predominance of the γ-Al_2_O_3_ phase, the increased number of structural defects, and the lower microhardness values (200 HV for E4, 177 HV for E5, and 182 HV for E6) led to a higher wear rate. These results indicate that the wear resistance of PEO coatings is determined not only by microhardness but also by the phase composition and the structural integrity of the layer.

The tribological performance of the PEO coatings, including the friction coefficient, wear-rate, and surface roughness, is presented in Table 4. The efficiency of friction coefficient and wear rate variation was evaluated to quantitatively compare the tribological behavior of the PEO coatings with the initial substrate. The efficiency (η) was calculated according to the method proposed by Yang et al. [35], using the following equation:

η = (X_substrate_ − X_coating_) × 100%/X_substrate_ (1)

where X_substrate_ and X_coating_ denote the friction coefficient or wear rate of the uncoated substrate and the coated sample, respectively. Positive values of η indicate an improvement (reduction in friction or wear), while negative values correspond to performance deterioration. The calculated efficiencies for all coatings are summarized in Table 4.

## 4. Conclusions

In this study, PEO coatings were successfully deposited on alumina electrolytes containing Al_2_O_3_ and SiO_2_ particles in an Al–Si alloy. The main results are as follows:

- Coatings E1 and E2 exhibited improved wear resistance compared to the initial sample (η_wear = +32% and +41%), and their microhardness increased to 230 HV(E1) and 173 HV (E2). This contributed to the reduction in the wear rate of these coatings. In particular, sample E2 showed a slightly improved surface morphology, with finer and more uniformly distributed pores.
- The E3 coating with added Al_2_O_3_ showed the most promising results: the wear rate decreased to 0.030 × 10^−3^ mm^3^/N·m, an improvement of approximately 96%. Although the microhardness increased only moderately (260 HV), the XRD results indicated that the α-Al_2_O_3_ phase was more pronounced in this coating. Therefore, the high wear resistance is explained mainly by the coating’s density and phase composition, not just by its hardness.
- No synergistic effect was observed in the E4–E6 systems with the combined Al_2_O_3_ + SiO_2_. The E4–E6 samples showed low wear resistance due to defects caused by particle agglomeration and unstable discharges. Although the coating produced in electrolyte E4 showed a smoother and more uniform surface appearance.

Overall, it was found that Al_2_O_3_ particles are the most effective additive in improving the wear properties of PEO coatings. Although the microhardness of all samples increased, the main factors for wear resistance were the formation of the α-Al_2_O_3_ phase and the structural density of the coating.

The thickness and surface roughness of the PEO coatings obtained in this study were lower compared to some systems in the literature, which is explained by the use of a high-frequency unipolar pulsed mode at 2000 Hz. Although this mode ensures stable micro-discharges, it also reduces the coating growth rate. Therefore, future work should focus on adjusting the frequency toward lower values, increasing the pulse on-time, extending the overall processing duration, and optimizing particle concentration to promote faster layer growth and improve structural uniformity while minimizing defects.

## Figures and Tables

**Table 2:** EDS analysis of the initial Al–Si substrate.

Element (wt.%)	O	Al	Si	Cr	Mn	Fe	Cu	Zn
Spectrum 1	2.24	58.83	9.95	0.91	6.18	19.65	2.01	0.22
Spectrum 2	2.25	93.96	2.07	0.00	0.00	0.13	0.77	0.83
Spectrum 3	2.29	67.88	28.21	0.00	0.00	0.00	0.95	0.67
Spectrum 4	3.51	70.68	6.07	0.52	4.19	12.45	2.10	0.47
Spectrum 5	2.13	72.18	13.09	0.14	1.53	9.53	0.75	0.64
Std. deviation	0.58	12.96	10.02	0.40	2.73	8.41	0.68	0.23

**Table 3:** EDS analysis of wear tracks on the PEO samples after tribological testing.

Sample	Spectrum	Al (wt.%)	O (wt.%)	Si (wt.%)	Fe (wt.%)	C (wt.%)
E1	1	56.59 ± 0.21	31.70 ± 0.16	6.50 ± 0.05	-	5.21 ± 0.32
	2	60.83 ± 0.23	28.02 ± 0.15	5.66 ± 0.05	-	5.49 ± 0.32
	3	56.49 ± 0.22	33.29 ± 0.17	4.84 ± 0.05	-	5.38 ± 0.33
E2	1	40.75 ± 0.05	45.77 ± 0.14	6.34 ± 0.02	0.60 ± 0.03	6.54 ± 0.20
	2	45.62 ± 0.15	45.96 ± 0.14	7.93 ± 0.05	0.49 ± 0.02	-
	3	51.73 ± 0.15	30.20 ± 0.17	11.38 ± 0.04	0.28 ± 0.04	6.31 ± 0.30
E3	1	12.95 ± 0.10	45.65 ± 0.11	5.26 ± 0.04	36.14 ± 0.21	-
	2	12.20 ± 0.04	46.42 ± 0.14	5.32 ± 0.06	36.06 ± 0.17	-
	3	2.87 ± 0.14	36.91 ± 0.16	1.04 ± 0.04	59.18 ± 0.18	-
E4	1	49.88 ± 0.19	39.14 ± 0.17	5.52 ± 0.05	-	5.45 ± 0.31
	2	83.17 ± 0.44	7.43 ± 0.12	2.75 ± 0.05	-	6.65 ± 0.48
	3	69.91 ± 0.33	13.51± 0.14	9.78 ± 0.07	-	6.81 ± 0.42
E5	1	50.62 ± 0.25	35.47 ± 0.21	7.01 ± 0.06	-	6.90 ± 0.40
	2	71.73 ± 0.54	5.33 ± 0.15	13.84 ± 0.13	-	9.10 ± 0.66
	3	61.18 ± 0.30	20.40 ± 0.13	10.43 ± 0.07	-	8.00 ± 0.39
E6	1	47.19 ± 0.03	46.43 ± 0.13	5.56 ± 0.02	0.82 ± 0.17	-
	2	60.05 ± 0.13	30.06 ± 0.17	3.76 ± 0.04	0.89 ± 0.04	5.24 ± 0.28
	3	40.46 ± 0.15	48.37 ± 0.18	5.89 ± 0.03	0.80 ± 0.07	4.48 ± 0.32

**Table 4:** Tribological parameters of the PEO coatings, including friction coefficient, wear rate efficiency, and surface roughness.

Sample	Coefficient of Friction	η (Friction) %	Wear Rate (10^−3^ mm^3^(Nm)^−1^)	η (Wear) %	Surface Roughness (R_a_)
Initial	0.446 ± 0.034	-	0.730 ± 0.141	-	-
E1	0.441 ± 0.027	+1.1	0.495 ± 0.100	+32.2	0.223 ± 0.016
E2	0.439 ± 0.022	+1.6	0.431 ± 0.248	+41.0	0.193 ± 0.039
E3	0.640 ± 0.030	−43.5	0.030 ± 0.002	+95.9	0.376 ± 0.038
E4	0.487 ± 0.053	−9.2	1.209 ± 0.777	−65.8	0.153 ± 0.008
E5	0.462 ± 0.045	−3.6	1.923 ± 0.512	−163.4	0.356 ± 0.027
E6	0.482 ± 0.065	−8.1	1.048 ± 0.396	−43.6	0.163 ± 0.028

## Data Availability

The original contributions presented in this study are included in the article. Further inquiries can be directed to the corresponding authors.
